# Optimization strategies for pulsed low‐dose‐rate IMRT of recurrent lung and head and neck cancers

**DOI:** 10.1120/jacmp.v15i3.4661

**Published:** 2014-05-08

**Authors:** Shengwei Kang, Jinyi Lang, Pei Wang, Jie Li, Muhan Lin, Xiaoming Chen, Ming Guo, Fu Chen, Lili Chen, Chang Ming Ma

**Affiliations:** ^1^ Radiation Oncology Department Sichuan Cancer Hospital Chengdu 610041 People's Republic of China; ^2^ Department of Radiation Oncology Fox Chase Cancer Center Philadelphia PA USA; ^3^ Department of Radiation Oncology Eye Ear Nose & Throat Hospital of Fudan University Shanghai People's Republic of China

**Keywords:** pulsed low‐dose‐rate radiotherapy (PLDR), IMRT, recurrent cancer, H&N cancer, lung cancer

## Abstract

Pulsed low‐dose‐rate radiotherapy (PLDR) has been proven to be a valid method of reirradiation. Previous studies of recurrent cancer radiotherapy were mainly based on conventional 3D CRT and VMAT delivery techniques. There are difficulties in IMRT planning using existing commercial treatment planning systems (TPS) to meet the PLDR protocol. This work focuses on PLDR using ten‐field IMRT and a commercial TPS for two specific sites: recurrent lung cancers and head and neck cancers. Our PLDR protocol requires that the maximum dose to the PTV be less than 0.4 Gy and the mean dose to be 0.2 Gy per field. We investigated various planning strategies to meet the PLDR requirements for 20 lung and head and neck patients. The PTV volume for lung cases ranged from 101.7 to 919.4 cm^3^ and the maximum dose to the PTV ranged from 0.22 to 0.39 Gy. The PTV volume for head and neck cases ranged from 66.2 to 282.1 cm^3^ and the maximum dose to the PTV ranged from 0.21 to 0.39 Gy. With special beam arrangements and dosimetry parameters, it is feasible to use a commercial TPS to generate quality PLDR IMRT plans for lung and head and neck reirradiation.

PACS number: 87.55.D‐

## I. INTRODUCTION

For recurrent cancers, most patients have undergone prior gross total resection, chemotherapy, and/or concurrent radiotherapy. After the initial treatment, once the patient has tumor progression in the previous radiation treatment field, the possibility exists that the patient is unable to undergo a second resection and conventional chemotherapy may have no effect in prolonging survival.^(1)^ Reirradiation may also cause severe damage to normal tissues near the target. It is a significant clinical challenge to improve median survival while minimizing the toxicity of treatment for recurrent cancers.

The dose‐rate effect in radiotherapy has been investigated for many years.^2^, ^3^ Pulsed low‐dose‐rate radiotherapy (PLDR) has been used in some institutions for treatment of recurrent cancer for several years.^4^, ^5^, ^6^, ^7^, ^8^, ^9^ The idea behind the PLDR technique is to take the advantages of both the hyperradiosensitivity of tumor cells below their transition doses above which cell repair mechanisms may be initiated to introduce radiation resistance, and the increased normal tissue repair at low‐dose rates.^(4)^ The way to achieve this is to divide a daily radiotherapy treatment into a number of subfractions (pulses), with each subfractional dose less than the tumor transition dose but greater than the normal tissue transition dose, so that the radiation repair is triggered in normal tissues but not in tumor cells. The radiation pulses are delivered at certain intervals to achieve an effective (or time‐averaged) low‐dose rate to maximize the normal tissue repair process. The application of PLDR in glioma and breast cancer has been reported by some investigators.^6^, ^7^ The existing PLDR technique using 3D conformal radiotherapy (3D CRT) divided the daily fractional 2 Gy dose into ten subfractions (pulses) with 3 min time intervals, creating a time‐averaged dose rate of 0.067 Gy/min.^(8)^ The dose rate of the accelerator was also reduced to 1 Gy/min. The cumulative dose delivered to the surrounding normal tissues may cause serious toxicities after reirradiation. Improved delivery methods may reduce the cumulative dose to organs at risk in PLDR.

It has been reported that it is possible to use one or two intensity‐modulated arcs to deliver PLDR plans to achieve better target coverage and normal tissue sparing for treatment of complex tumors.^9^, ^10^, ^11^ The PLDR treatment of recurrent cancers using dynamic arc therapy delivers 0.2 Gy to the target per arc with a 3 min interval. Rong et al.^(10)^ demonstrated that with special machine parameters, a tomotherapy plan could be delivered within the 3 min time interval to meet the PLDR treatment requirement with a 3 min interval between fields. Tyagi et al.^(11)^ showed that previously treated glioblastoma multiforme (GBM) cases could be replanned using volumetric‐modulated arc therapy (VMAT) with a single 160° arc to deliver 0.2 Gy pulses on the new Elekta accelerator (Elekta AB, Stockholm, Sweden). Ma et al.^(9)^ implemented the PLDR technique using the Varian RapidArc technique (Varian Medical Systems, Palo Alto, CA). They used two arcs to produce high‐quality PLDR plans to further spare nearby critical structures, which could be delivered five times to achieve a daily dose of 2 Gy, resulting in a time‐averaged dose rate of 0.067 Gy/min. However, for some hospitals in the United States and a large number of hospitals in developing countries, advanced dynamic arc therapy techniques such as the Varian RapidArc or Elekta VMAT are not available. Therefore, for complex recurrent cancers, IMRT may be the only choice to deliver PLDR treatment at these institutions.

A technical difficulty in PLDR treatment planning using advanced radiotherapy techniques is to ensure the delivery of a complex plan (i.e., the 0.2 Gy dose pulse) within the 3 min time interval because more complex plans may require more MLC segments, more monitor units, and longer delivery time. Ideally, the total beam‐on time should be minimal so that there is sufficient time between the individual dose pulses to avoid the possibility of accumulation of individual pulses to exceed the tumor transition dose and to trigger the cell repair process. The “optimal” interval for PLDR is unknown, but for practical reasons a 3 min interval will result in a 30 min treatment time that is tolerable by most recurrent patients. For conventional RT with a 300‐600 MU/min dose rate, the actual beam‐on time to deliver 0.2 Gy is only a few seconds. For VMAT and RapidArc, a full arc will take >1 min beam‐on time with <2 min left between the individual pulses. For tomotherapy, the beam‐on time could be close to 3 min for large targets, and the dose delivery becomes almost continuous and the cumulative dose could exceed the transition dose after a few pulses. For our ten‐field IMRT delivery, the beam‐on time for a single field is typically <1 min. However, it would be impossible to deliver a complex IMRT plan with multiple gantry angles (fields) within the 3 min interval. Therefore, it is necessary to deliver 1 field/arc per pulse for PLDR if the IMRT/VMAT delivery technique is used.

A simple solution for PLDR using IMRT is to design a plan with ten gantry angles (fields) with each field delivering 0.2 Gy in a 3 min interval.^(12)^ Problems arise when optimizing the treatment plan using existing commercial treatment planning systems, which were designed to produce a conformal and uniform dose distribution to the target volume with all of the fields used (i.e., after the entire plan is delivered); the target dose distribution per field can be very heterogeneous due to beam attenuation (in the beam direction) and intensity modulation (in the directions perpendicular to the beam axis). This represents a unique problem for PLDR planning with multiple arc/fields because the maximum dose per pulse (arc/field), which is not a dose constraint in an existing commercial TPS, may exceed the tumor transition dose. The challenge is to generate IMRT plans using an existing TPS to meet the PLDR treatment criteria.

In this work, the application of the Varian Eclipse treatment planning system (Varian Medical Systems) was used for two complex treatment sites: lung and head and neck. To achieve the specific treatment planning objectives for PLDR, various planning methods were investigated including beam angle optimization, dose constraints, and reference structure design, as well as other auxiliary skills.

## II. MATERIALS AND METHODS

### A. Patient cases

In this study, ten lung cases and ten head & neck cases were investigated. The cases included cancers of the right or left lung, in or around the mediastinum, and in the tongue, oropharynx, or larynx. The PTV volume was delineated on CT images. Table 1 lists the PTV volumes for the ten lung cases and ten head and neck cases. The energy used for the treatment was 6 MV for both lung and head and neck sites. The total prescription dose for the lung site was 60 Gy, which was delivered in 30 fractions resulting in a daily 2 Gy fractional dose. The prescription dose for head & neck was 70 Gy delivered in 35 fractions and 2 Gy per fraction. The dose distributions were normalized to ensure that 95% of the target volume would receive 100% of the prescription dose. We also compared IMRT plans with 3D CRT plans for PLDR treatments to evaluate the applicability of the two techniques in complex PLDR planning.

**Table 1:** PTV volumes for the ten lung patients and ten head and neck patients investigated in this work

*Patient Number*	*1*	*2*	3	*4*	*5*	*6*	*7*	*8*	*9*	*10*
Lung	269	101.7	188.1	361	171	366	426	919.4	401	486
	(C)	(C)	(C)	(C)	(C)	(R)	(L)	(R)	(L)	(L)
Head & Neck	95.7	171	280.8	128.4	282.1	145.3	92.6	66.2	157.6	246.5

Note that the collimator angle and couch angle is 0° for all plans.

C=central lung; L=left lung; R=right lung.

### B. Treatment planning

Most medical physicists and dosimetrists are familiar with conventional, 3D CRT, and IMRT treatment planning skills. However, the PLDR protocol also has additional requirements. For example, the maximum dose per pulse and the effective low dose rate for the entire treatment cannot be automatically optimized by the optimizers of existing commercial treatment planning systems. During the planning process, we have to manually optimize the beam angles and many other parameters in order to meet the PLDR plan acceptance criteria. Sometimes we need to contour additional reference structures and define objectives such as the constraint of maximum dose for PLDR optimization (see below and also the Discussion section for more details). These optimization techniques appear similar to those used for regular IMRT planning, but with different objectives.

In this work, the Varian Eclipse system (version 10.0) was used to generate all of the IMRT and 3D conformal plans for PLDR treatments, which includes a function that allows the user to compute the dose distributions of individual fields for a multifield IMRT plan. The AAA algorithm was used for dose calculation with the heterogeneity correction switched on. The sliding window method was used for the treatment delivery on Varian iX accelerators (Varian Medical Systems). We used 6 MV photon beams for all lung and head and neck treatment plans. For IMRT plans, ten gantry angles/fields were chosen based on the target and critical structure geometry to achieve the planning goals. The total dose per fraction was 2 Gy. The individual gantry angles/fields were delivered in 3 min time intervals, which resulted in an effective dose rate of 0.067 Gy/min. An important plan acceptance criterion for PLDR is that the maximum target dose for any particular gantry angle/field is less than 0.4 Gy, which was selected for our PLDR treatment planning protocol as a reasonable dose limit to avoid triggering cell repair mechanisms for those tumor cells that exhibit hyperradiosensitivity.^13^, ^14^, ^15^ There was no real limit on the minimum dose in the target volume per gantry angle/field since there was no lower limit on the minimum target dose rate. However, as any “cold spots” in the target volume from a particular gantry angle/field would have to be compensated by “hot spots” from other gantry angles/fields, the target dose heterogeneity (the difference between the maximum dose and the minimum dose) should be kept as low as possible.

For centrally located lung cases, ten gantry angles were arranged uniformly for most cases and the angles were separated by approximately 35°. Applying this angle arrangement, a con‐formal and uniform dose distribution could be easily obtained for most centrally located lung tumors and meet the dosimetric criteria of our PLDR protocol.

For peripheral lung cases, gantry angles were arranged in an arc by nearly the same angle intervals surrounding the target on one side. This arrangement of beam angles could achieve a conformal and uniform dose distribution to the target by each gantry angle simultaneously minimizing doses to the opposite normal lung.

If some areas inside the target contained hot spots of greater than 0.35 Gy from one gantry angle, an attempt was made to adjust the beam angle by a few degrees and/or draw reference structures, such as inner ring and outer ring of the target, to reduce dose heterogeneity in these areas. For some particular cases, the adjustment of several beam angles might be necessary to obtain an angle optimization where the maximum dose‐to‐target volume could be kept under 0.4 Gy for each individual field.

In 3D CRT planning, we used three to five beams passing through one side of the lung for peripheral lung cases. For centrally located lung cases, we used two opposed beams, one anterior and the other posterior.

For head and neck planning, equally divided beam angles worked well for some PLDR plans. However, for other cases, certain beam angles should be avoided that pass through the spinal cord or other critical organs because the PLDR technique is mainly used for recurrent cancers. It was found that hot spots may exist inside the PTV volume when the target was too close to the teeth. Subsequently, it was necessary to adjust the beam angle, which originally passed through the teeth, to a new direction. For peripheral targets, the ten gantry angles were approximately uniformly arranged in a partial arc.

For 3D conformal planning of the head and neck cases, two opposed lateral beams were applied to achieve a mean dose of 0.2 Gy at 3 min intervals by dividing the total monitor units (MU) accordingly.

## III. RESULTS

### A. Lung

**Figure 1:**
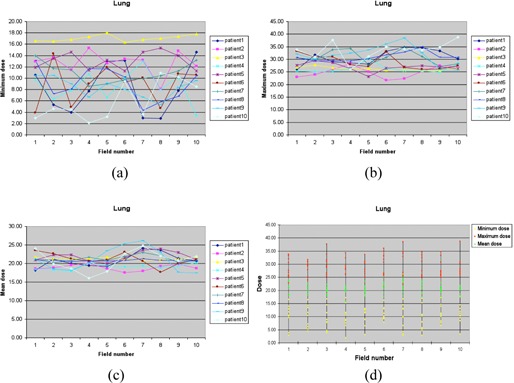
The minimum dose (a), maximum dose (b), and mean dose (c) of the PTV of each gantry angle/field for ten lung cases, and the distribution (d) of the minimum dose, maximum dose, and mean dose (unit: cGy).

**Figure 2:**
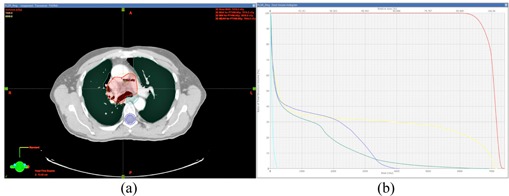
Isodose lines (a) (66 Gy and 70 Gy, respectively) and DVHs (b) of the PTV and critical structures of Patient 3.

For the lung site, five cases were centrally located lung tumors, three cases were cancers in the left lung, and two cases were cancers in the right lung. The maximum PTV dose per gantry angle/field for the ten cases ranged from 0.22 Gy to 0.39 Gy, all of which met the PLDR criterion of $D_{\text{max}}<0.4$ Gy. The mean PTV dose per gantry angle/field ranged from 0.19 to 0.22 Gy. Figure 1 shows the minimum dose (Fig. 1(a)), maximum dose (Fig. 1(b)), and mean dose (Fig. 1(c)) for the PTV volume of each gantry angle/field for all ten cases. Our greatest concern about IMRT‐based PLDR treatments is the maximum dose for each gantry angle. Seen from (Fig. 1(b)), Patient 2 and Patient 3 are better than other plans, with the maximum dose for all gantry angles less than 0.3 Gy. The plan parameters for Patient 3 (yellow points) demonstrate that it is a good plan for PLDR, with the minimum dose higher than 0.16 Gy, the maximum dose less than 0.28 Gy, and the mean dose at 0.21 Gy for all the gantry angles. For the other plans, the PTV was covered by less than 0.1 Gy for some gantry angles. However, the minimum dose to the PTV for each gantry angle is only a soft constraint for PLDR planning, since there is no lower limit on the minimum effective dose rate. The minimum dose, maximum dose, and mean dose for each gantry angle of the ten cases is summarized in Fig. 1(d). Figure 2 shows the dose distributions and DVHs of Patient 3.

### B. Head and neck

**Figure 3:**
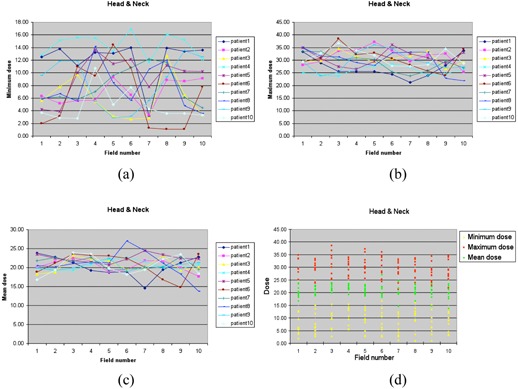
The minimum dose (a), maximum dose (b), and mean dose (c) of the PTV of each gantry angle/field for ten head and neck cases, and the distribution (d) of the minimum dose, maximum dose, and mean dose (unit: cGy).

**Figure 4:**
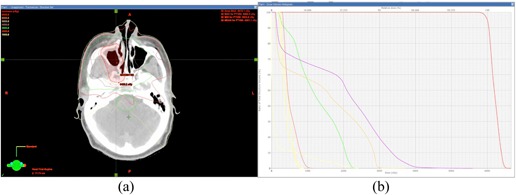
Isodose lines (a) (10 Gy to 65 Gy in 5 Gy steps) and (b) DVHs of the PTV and critical structures of Patient 1.

Ten head and neck cases were investigated for this study. The maximum PTV dose per gantry angle ranged from 0.21 to 0.39 Gy, all of which met the requirement of the maximum dose limit of less than 0.4 Gy. The mean PTV dose per gantry angle ranged from 0.14 to 0.25 Gy. The minimum (Fig. 3(a)), maximum (Fig. 3(b)), and mean (Fig. 3(c)) PTV doses for each gantry angle are shown in Fig. 3. Patient 4 showed better PTV dose coverage than the other plans, and the maximum dose for each gantry angle was less than 0.3 Gy. Figure 3(d) summarizes the minimum, maximum, and mean doses per gantry angle for all ten plans investigated. Figure 4 shows the dose distribution and DVH of Patient 1.

## IV. DISCUSSION

Previous publications on PLDR were mainly based on conventional radiation therapy^5^, ^6^, ^7^ and dynamic arc therapy technology.^9^, ^10^, ^11^ A daily dose of 2 Gy per fraction could be easily divided into ten 0.2 Gy subfractions in conventional radiotherapy, since the dose to the reference point of the target volume and the MUs used in a subfraction have a linear relationship with each other. However, for some complex recurrent cancers, there are many critical structures surrounding the target, in which case the conventional treatment techniques are not suited for PLDR. Further studies have been focused on developing dynamic arc therapy techniques for PLDR dose delivery using a dual‐arc plan delivered five times. The advantage of using dynamic arc therapy for PLDR treatments is that it could reduce the dose to the critical structures. Recent publications demonstrated that rotational therapy plans could handle most clinical recurrent cancers for PLDR treatments of different cancer sites.^9^, ^10^, ^11^

Although intensity‐modulated dynamic arc therapy is more advantageous than IMRT in PLDR dose delivery,^(16)^ some hospitals may not have an advanced tomotherapy unit, or a Varian RapidArc or a state‐of‐the‐art Elekta VMAT accelerator. In this study, we proposed to use ten gantry‐angle IMRT plans for PLDR treatments. The existing commercial treatment planning systems are not specifically designed for PLDR treatment planning. Dose distributions and isodose curves of a regular IMRT plan are more dependent on the relative direction of the incident beams to the nearby critical structures. In order to develop practical guidelines for treatment planning of PLDR treatments using regular IMRT techniques, we chose two specific cancer sites for this retrospective treatment planning and dosimetry comparison study.

The first site we selected for this study was lung cancer, which has a significant density difference between the target volume and normal lung tissues. Of the selected five centrally located lung cancers, ten beam angles were separated by an approximately 35° interval, which showed acceptable results for most centrally located lung cases. Since the target volume of a centrally located lung cancer was in the middle of the human body, a uniform beam arrangement could successfully produce a 0.2 Gy dose contribution to the PTV for each gantry angle with the maximum dose of each gantry angle (or each 3 min interval) being kept under 0.4 Gy. For peripheral lung cancers, the ten beam angles were selected close to the target side of the lung at intervals of 20° to 30°. The actual beam angles could be adjusted according to the situation. The maximum dose to the target volume before and after the adjustment of the three gantry angles is shown in Table 2. The improvement was significant with this approach. Sometimes, however, it was insufficient to generate a high‐quality plan with only the beam angle adjustment for PLDR treatment planning. In order to solve this problem, our experience was to draw reference structures inside and/or outside the PTV with specially assigned dose constraints during treatment optimization. An example is Patient 9: both angle adjustment and two reference rings were used to reduce the maximum dose to the PTV for field1, field3, field6 and field7. It can be seen from Table 3 that the maximum doses for these fields were reduced to less than 0.35 Gy; however, for field4, the maximum dose actually increased with the beam angle adjustment, and was successfully minimized to less than 0.35 Gy by applying the reference rings.

**Table 2:** Maximum dose to the PTV before and after the angle adjustment of the three gantry angles

	*Field6*	*Field7*	*Field8*
Dose 1^a^	34.2	36.1	32.6
Dose 2^b^	33.2	34.8	34.6

a Dose 1 is before the adjustment.

b Dose 2 is after the adjustment.

**Table 3:** Maximum doses to the PTV before and after beam angle adjustments and reference structures that were used for five angles

	*Fieldl*	*Field3*	*Field4*	*Field6*	*Field7*
Dose 1^a^	35.4	35.5	34.3	39.5	36.3
Dose 2^b^	30.6	34.1	35.3	30.8	34.3
Dose 3^c^	30.6	33.8	34.3	30.8	34.8

a Dose 1 is before the beam angle adjustment.

b Dose 2 is after the beam angle adjustment.

c Dose 3 is after the reference structure was used.

**Figure 5:**
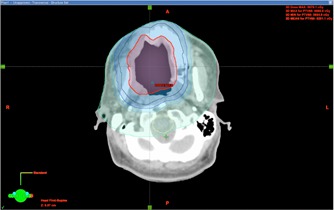
The reference ring structures that were used to minimize hot spots inside the target volume. The red contour is the PTV. Four reference rings were used in this case.

The second site for this study was head and neck cancer, which usually contains cavities and bones adjacent to the target volume. It was found useful to arrange the ten incident beam angles surrounding the target volume as a circle or a sector at an interval of 10° to 30°. This beam angle arrangement has been shown to be suitable for most cases, although sometimes reference structures may be needed to reduce the hot spots inside the PTV caused by nearby bones or teeth. Figure 5 shows how the reference ring structures were used to minimize hot spots appearing inside the target volume due to the effects of nearby teeth and bones. The maximum dose was constrained by the rings in order to prevent any possible hot spots inside nearby teeth and bones from extending into the target volume. Since the PLDR treatment is developed mainly for recurrent cancers, the spinal cord and brainstem for patients with recurrence cannot receive a dose as high as that for the initial IMRT treatment. If uniform beam angles were used for planning, the spinal cord and brainstem might receive high doses because every incident beam would pass through the spinal cord or brainstem. Therefore, nonuniform beam angles were selected in order to avoid the beams passing through the spinal cord and brainstem, if possible.

**Figure 6:**
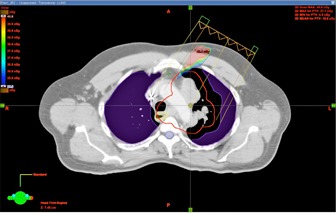
Example of a particular beam angle that delivers a maximum dose to the PTV between 35 cGy and 40 cGy for Patient 10.

Hyperradiosensitivity has been shown at small doses below some transitional doses around 0.5 Gy or at very low dose rates.^(3)^ In this research, the ideal maximum dose of an individual field (or a 3 min interval) was less than 0.35 Gy at the hot spot of the target volume. However, it was not possible to ensure that this ideal dose limit could be met for every gantry angle/field and for every PLDR patient. For the patient cases investigated, five head and neck patients showed maximum doses more than 0.35 Gy for a gantry angle. To maximize the hyperradiosensitivity effect, we can set this gantry angle as the last one to deliver to minimize its potential to trigger cell repair. There was a lung patient treatment plan that had three gantry angles delivering more than 0.35 Gy but less than 0.4Gy, which is the maximum dose allowable for PLDR treatments based on our clinical acceptance criteria. In that case, we could arrange these three gantry angles to be the last three to be irradiated or switch to intensity‐modulated dynamic arc therapy for PLDR treatments. Figure 6 shows an example of a gantry angle that delivers a maximum dose to the PTV more than 0.35 Gy for Patient 10. There are hot spots in the proximal portion and cold spots in the distal portion of the target volume relative to the incident beam direction.

**Figure 7:**
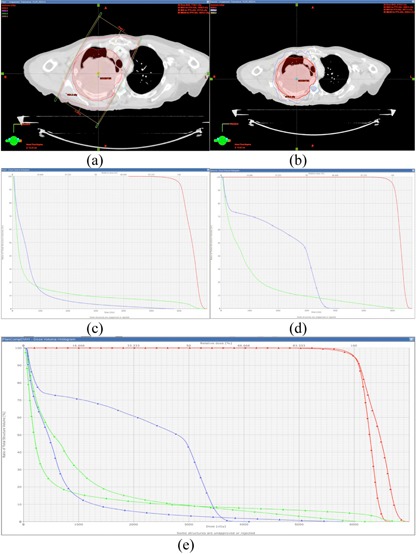
The isodose distributions of a 3D CRT plan (a) and an IMRT plan (b), the corresponding DVHs of the two plans (c) and (d), and the comparison of the DVHs of the two plans (e) for a lung cancer patient.

**Figure 8:**
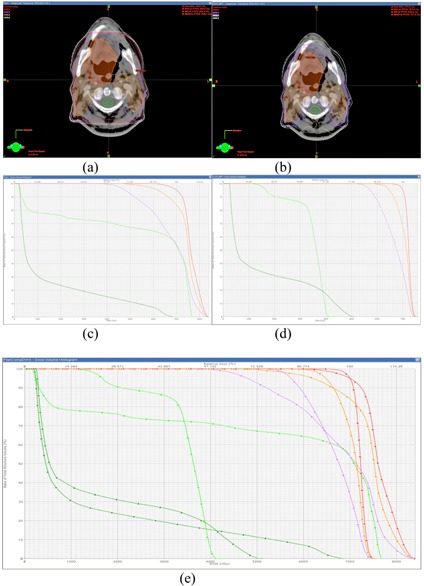
The isodose distributions of a 3D CRT plan (a) and an IMRT plan (b), the corresponding DVHs of the two plans (c) and (d), and the comparison of the DVHs of the two plans (e) for a head and neck patient.

In addition, the dosimetry differences between conventional treatments and IMRT have been investigated for PLDR patients. Figure 7 shows the isodose distribution and DVHs of a lung cancer patient. Figures 7(a) and (b) are isodose curves of a 3D CRT plan and an IMRT plan, respectively. Figures 7(c) and (d) are DVHs of the two plans, respectively. Figure 7(e) compares the DVHs of the two plans. For the 3D CRT plan of this lung patient, we set the beams to avoid any of them passing through the spinal cord, but the maximum dose to the cord was still nearly 60 Gy since the target and the cord were very close to each other. The maximum dose to the spinal cord for the IMRT plan was less than 40 Gy, which was much lower than the 3D CRT plan. Figure 8 further compares the isodose distribution and DVHs between 3D CRT and IMRT for a head and neck patient. The maximum doses to the spinal cord and brainstem for the 3D CRT plan were much higher than those for the IMRT plan. These two examples demonstrated that, for special target shapes and complex relationships between the target and critical structures, intensity‐modulated dynamic arc therapy and IMRT may be the only choices for PLDR of recurrent patients.

## V. CONCLUSIONS

Manually optimized ten gantry‐angle IMRT plans generated using commercial treatment planning systems are adequate for PLDR treatments of lung and head and neck recurrent cancers. Compared with 3D CRT treatment plans, IMRT plans provide better sparing of critical structures for cases with complex relationships between the target and critical structures, which is essential for recurrent cancer treatment. In addition to dual‐arc, intensity‐modulated arc radiotherapy, IMRT can be a valid option for the PLDR treatment of complex lung and head and neck recurrent cancers.

## References

[1] Hess KR , Wong ET , Jaeckle KA , et al. Response and progression in recurrent malignant glioma. Neurooncol. 1999;1(4):282–88. 10.1215/15228517-1-4-282 PMC1920759 11550320

[2] Hall EJ and Brenner DJ . The dose‐rate effect revisited: radiobiological considerations of importance in radiotherapy. Int J Radiat Oncol Biol Phys. 1991;21(6):1403–14. 1938548 10.1016/0360-3016(91)90314-t

[3] Steel GG , Deacon JM , Duchesne GM , Horwich A , Keiland LR , Peacock JH . The dose‐rate effect in human tumour cells. Radiother Oncol. 1987;9(4):299–310. 3317524 10.1016/s0167-8140(87)80151-2

[4] Ma CM , Luxton G , Orton CG . Point/Counterpoint: Pulsed reduced dose rate radiation therapy is likely to become the treatment modality of choice for recurrent cancers. Med Phys. 2011;38(9):4909–11. 21978035 10.1118/1.3583794

[5] Cannon GM , Tome WA , Robins HI , Howard SP . Pulsed reduced dose‐rate radiotherapy: case report, a novel retreatment strategy in the management of recurrent glioblastoma multiforme. J Neurooncol. 2007;83(3):307–11. 17252184 10.1007/s11060-007-9329-z

[6] Adkison JB , Tome W , Seo S , et al. Reirradiation of large recurrent glioma with pulsed reduced dose‐rate radiation therapy. Int J Radiat Oncol Biol Phys. 2011;79(3):835–41. 20472350 10.1016/j.ijrobp.2009.11.058

[7] Richards GM , Tome WA , Robins HI , et al. Pulsed reduced dose‐rate radiotherapy: a novel locoregional retreatment strategy for breast cancer recurrence in the previously irradiated chest wall, axilla, or supraclavicular region. Breast Cancer Res Treat. 2009;114(2):307–13. 18389365 10.1007/s10549-008-9995-3

[8] Tome WA and Howard SP . On the possible increase in local tumour control probability for gliomas exhibiting low dose hyper‐radiosensitivity using a pulsed schedule. Br J Radiol. 2007;80(949):32–37. 16945935 10.1259/bjr/15764945

[9] Ma CM , Lin MH , Dai XF , et al. Investigation of pulsed low dose rate radiotherapy using dynamic arc delivery techniques. Phys Med Biol. 2012;57(14):4613–26. 22750648 10.1088/0031-9155/57/14/4613

[10] Rong Y , Paliwal B , Howard SP , Welsh J . Treatment planning for pulsed reduced dose‐rate radiotherapy in helical tomotherapy. Int J Radiat Oncol Biol Phys. 2011;79(3):934–42. 20884127 10.1016/j.ijrobp.2010.05.055

[11] Tyagi N , Yang K , Sandhu R , et al. External beam pulsed low dose radiotherapy using volumetric modulated arc therapy: planning and delivery. Med Phys. 2013;40(1):011704. 23298074 10.1118/1.4769119

[12] Price RA , Kuritzky N , Lin T , Ma C . Pulsed reduced dose‐rate intensity modulated radiotherapy (IMRT) delivery for use in the high dose re‐irradiation setting [abstract]. Med Phys. 2009;36(6):2553.

[13] Joiner MC , Marples B , Lambin P , Short SC , Turesson I . Low‐dose hypersensitivity: current status and possible mechanisms. Int J Radiat Oncol Biol Phys. 2001;49(2):379–89. 11173131 10.1016/s0360-3016(00)01471-1

[14] Marples B and Joiner MC . The elimination of low‐dose hypersensitivity in Chinese hamster V79‐379A cells by pretreatment with X rays or hydrogen peroxide. Radiat Res. 1995;141(2):160–69. 7838954

[15] Short SC , Kelly J , Mayes CR , Woodcock M , Joiner MC . Low‐dose hypersensitivity after fractionated low‐dose irradiation in vitro. Int J Radiat Biol. 2001;77(6):655–64. 11403705 10.1080/09553000110041326

[16] Lin MH , Price RA , Li JS , Kang SW , Li J , Ma CM . Investigation of pulsed IMRT and VMAT for re‐irradiation treatments: dosimetric and delivery feasibilities, Phys Med Biol. 2013;58(22):8179–96. 24200917 10.1088/0031-9155/58/22/8179

